# Anesthetic and Intensive Care Approaches Following Radical Pneumonectomy: A Short Review of Patient Management and a Case Report

**DOI:** 10.7759/cureus.64786

**Published:** 2024-07-18

**Authors:** Bogdan I Vintila, Alina S Bereanu, Ioana R Codru, David Achim, Stefan A Bancila, Mihai Sava

**Affiliations:** 1 Anesthesia and Critical Care, County Clinical Emergency Hospital, Sibiu, ROU; 2 Anesthesia and Critical Care, Faculty of Medicine, Lucian Blaga University, Sibiu, ROU; 3 Pharmacology, Faculty of Medicine, Lucian Blaga University, Sibiu, ROU; 4 Thoracic Surgery, County Clinical Emergency Hospital, Sibiu, ROU

**Keywords:** pulmonary embolism, picco, rrt, hemorrhagic shock, anesthesia for pneumonectomy

## Abstract

Around the world, lung cancer is the leading cause of cancer-related death and the most commonly diagnosed cancer. In the early stages, surgery is the preferable therapeutic strategy. We present the case of a male patient aged 49 years diagnosed with non-small cell lung cancer of the left lower lobe who was referred for a radical left pneumonectomy. After careful preoperative preparation, the surgery was proceeded with. During the surgery, the patient needed bronchoscopy for the aspiration of the trachea and bronchial tree; after the aspiration procedure, an intraoperative massive hemorrhage started, with shock and ventricular tachycardia. Nine days after surgery, the patient developed a pulmonary embolism and returned to the ICU. The patient benefited from transfusion, intrathoracic cardiac compressions, pulse index continuous cardiac output (PiCCO), renal replacement therapy (RRT), anticoagulation, and intensive care. After a complicated clinical course, the patient is discharged, and after more than 18 months, the patient comes regularly for follow-up consultation in good health.

## Introduction

Lung cancer is the most frequently diagnosed cancer and the leading cause of cancer-related deaths worldwide. It is the second most commonly diagnosed cancer, with an estimated two million new cases and 1.8 million deaths yearly [1].

Significant progress has been made in lung cancer treatment over time, improving patient prognoses. Often, lung cancer requires multiple treatment options, including surgery, radiotherapy, and systemic therapies [2]. In the case of early-stage lung cancer, thoracic surgery is considered the standard treatment, and with the development of video-assisted thoracoscopic surgery (VATS), the perioperative mortality and long-term survival rates have improved [3].

With regard to early recovery after surgical procedures, the patient's general health is assessed, which may involve various tests such as pulmonary function tests, electrocardiograms, echocardiograms, imaging studies, and laboratory tests. A comprehensive anesthesia plan is also developed, encompassing aspects of patient care such as counseling, nutrition, smoking cessation, alcohol management, pre-anesthetic medication management, and postoperative care [4,5].

The anesthetic technique necessitates special consideration, including invasive blood pressure, heart rate, oxygen saturation, end-tidal carbon dioxide, and urine output monitoring. Essential components involve intravenous access, the placement of an epidural catheter for pain management, and lung isolation using a double-lumen tube or bronchial blocker. Anesthesia maintenance uses an air/oxygen mix, volatile anesthetics, and lung-protective ventilation strategies. Proper precautions should be taken before placing the patient in the lateral position [6-8].

Managing a patient during one-lung ventilation (OLV) poses several challenges due to respiratory physiological changes. These include alterations in respiratory mechanics, such as reduced functional residual capacity, atelectasis, increased resistance, and air trapping. Additionally, changes in oxygenation, such as right-to-left shunt and blood flow redistribution, as well as changes in hemodynamics, like increased pulmonary vasoconstriction and increased load on the right ventricle due to hypoxic pulmonary vasoconstriction, need special consideration [9].

Postoperative care involves using a multimodal pain management approach, including regional analgesia, to minimize or avoid the use of opioids and to reduce postoperative pulmonary complications. Following lung surgery, common reasons for ICU admission include respiratory failure, infections, acute respiratory distress syndrome, lobar collapse, and pulmonary embolism. Other reasons for ICU admission include renal failure, cardiac arrest, arrhythmia, and bleeding. Essential strategies of postoperative care include promoting bronchial hygiene, early patient mobilization, physiotherapy, early extubation, minimizing the risk of healthcare-associated infections, maintaining proper fluid balance, maintaining normal cardiac output, avoiding nephrotoxic medications, providing necessary nutritional support, and ensuring appropriate follow-up after the surgery [10-13].

Advancements in surgical techniques, anesthetic agents, and the accumulation of scientific knowledge and guidelines have significantly improved the safety of lung surgery. Through our case report, we aim to contribute to the expanding knowledge base and improve lung resection outcomes by prioritizing patient safety and reducing the incidence of complications.

## Case presentation

We report the case of a 49-year-old Caucasian male diagnosed with non-small cell lung cancer in the left lower lobe, with a history of bronchial emphysema, chronic obstructive pulmonary disease, right branch block, and a history of smoking. Before being scheduled for surgery, the patient underwent evaluation by a multidisciplinary team comprising physicians from the pneumology, thoracic surgery, oncology, anesthesia, and intensive care departments.

The bronchoscopy indicated an overall appearance, suggesting chronic bronchitis and mucous secretions. No abnormal masses were found in the right bronchus. However, on the left bronchus, there was a sessile, budding tumor formation covered with false membranes, which were fragile and causing almost complete obstruction of the lower left lobe. The bronchial biopsy and histopathological examination confirmed the presence of non-small cell lung cancer.

Spirometry results indicated a distal obstructive syndrome and a moderate alteration of gas transfer through the capillary alveolar membrane. Plethysmography demonstrated an increase in total lung capacity to 8.50L (120%) and an increase in residual volume to 3.42L (158%).

**Figure 1:**
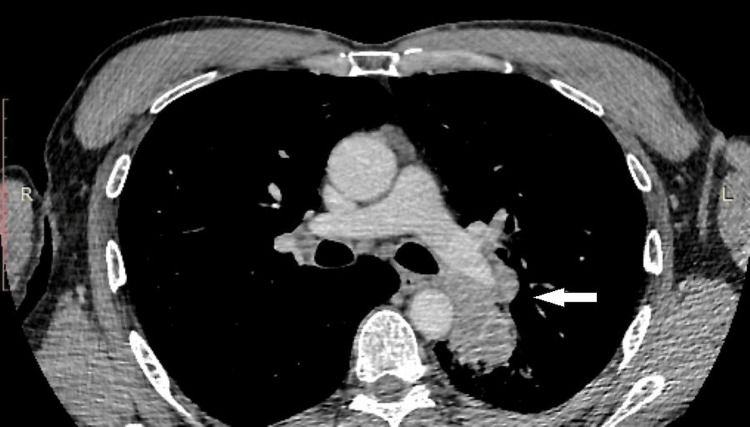
The axial section of the thoracic preoperative CT scan showing tumor relationship to the main pulmonary artery, descending aorta, and left main bronchus. CT: computed tomography

**Figure 2:**
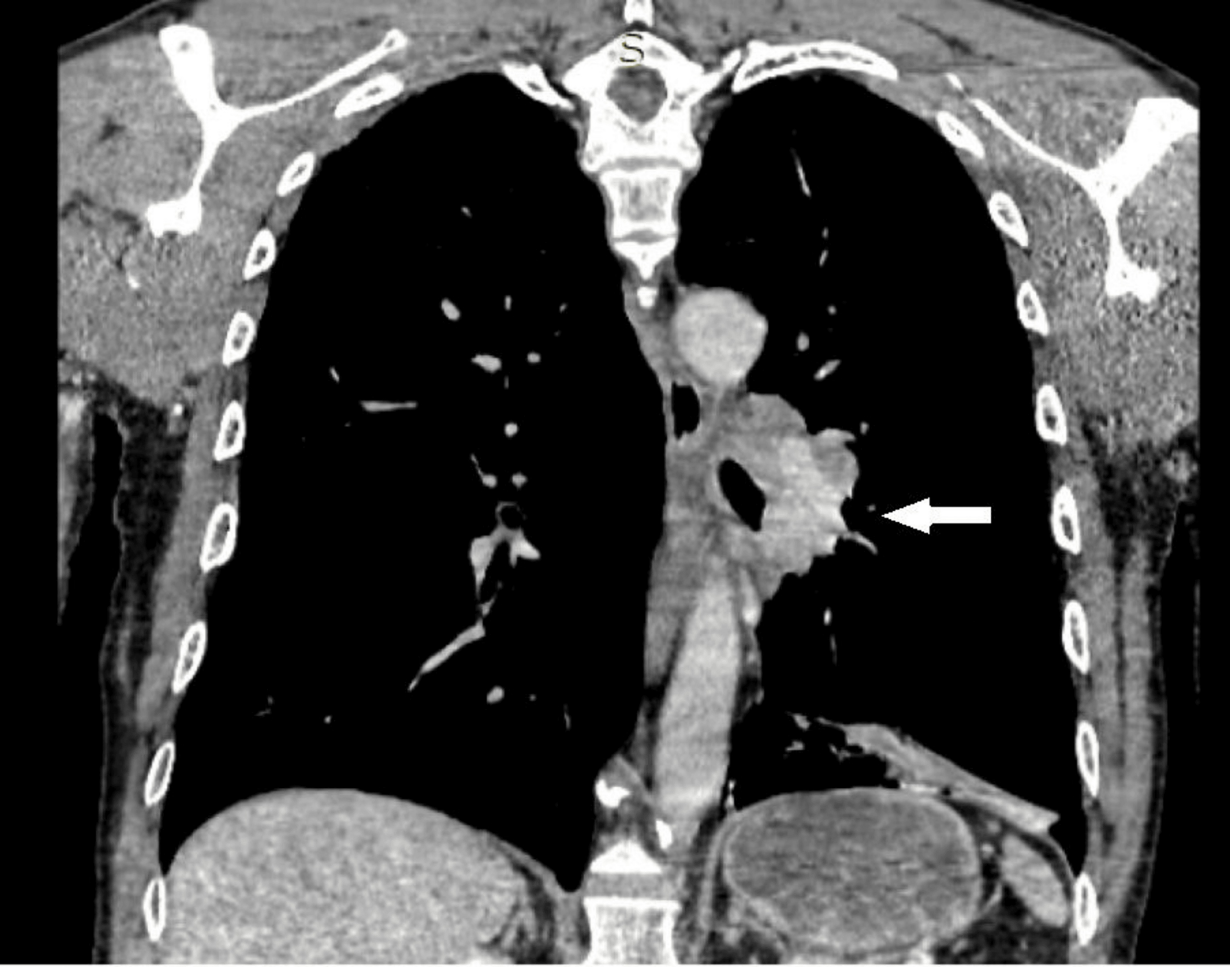
The coronal section of the preoperative thoracic CT scan showing tumor relationship to the left hilum. CT: computed tomography

The whole-body computed tomography (CT) scan identified a hilar and infra-hilar iodophilic tissue mass in the lower left lobe measuring approximately 5/6.7 cm. The mass obstructed the left lower lobar bronchus, with partial atelectasis. Additionally, the mass was in contact with the left lateral wall of the thoracic aorta at a craniocaudal distance of approximately 6 cm (Figure 1 and Figure 2).

The preoperative cardiologic evaluation revealed stable effort angina pectoris, right branch block, and sinus tachycardia. The echocardiography showed the following results: normal heart cavities without any motion abnormalities, left ventricular ejection fraction (LVEF) of 60%, normal heart valves, absence of pulmonary hypertension, and an aneurysm of the interatrial septum without any shunt. Based on the evaluation, nebivolol and trimetazidine were prescribed. The results of the blood laboratory tests, including coagulation test, complete blood count, creatinine, and liver function tests, were all within normal ranges, and no further investigations were required. Following the preoperative assessment and consultation with an oncologist, a left pneumonectomy was recommended.

Prior to the scheduled surgery, the Ag SARS-CoV2 test came back positive, leading to the postponement of the procedure until the patient achieved complete respiratory recovery. The patient chose to be discharged from the hospital rather than being transferred to the infectious disease department. Following an 11-day interval, the patient returned for the surgery without exhibiting any acute symptoms or significant changes in the blood tests.

Two units of crossmatched blood were set aside at the transfusion unit prior to the surgery. The patient was brought to the operating room; his vitals were stable, and he received a premedication of 2 mg of midazolam. An epidural catheter was positioned at the T10 level, and an arterial line was inserted for continuous monitoring and periodic blood gas analysis.

Following a standard induction with sufentanil (15 mcg), propofol (100 mg), and succinylcholine (100 mg), the left double-lumen tracheal tube was inserted without complications. General anesthesia was maintained using sufentanil (total dose 240 mcg), rocuronium (total dose 200 mg), sevoflurane, and a low-dose norepinephrine infusion (0.1-0.2 mcg/kg/min). Subsequently, prior to positioning the patient in the lateral decubitus position, a central line was inserted into the internal jugular vein.

A left posterolateral thoracotomy was performed to access the pleural cavity through the fifth intercostal space. While dissecting and sectioning the triangular ligament, a drop in the patient's SpO2 was observed, leading to a temporary cessation of the dissection. Subsequently, after a rapid assessment of the situation, consensus was reached to safely reposition the patient in the supine position for the replacement of the endotracheal tube with a single-lumen tube for intraoperative bronchoscopy. Bronchoscopic examination revealed adherent viscous secretions obstructing the lumens of the main bronchi, which were promptly aspirated.

Following the stabilization of the patient's oxygenation and ventilation, significant bleeding from the pulmonary hilum was observed. During the effort to achieve surgical hemostasis, the patient experienced hemorrhagic shock and ventricular tachycardia, necessitating defibrillation and internal cardiac massage. Resuscitation efforts included the administration of blood, fresh frozen plasma, fluids, and vasopressor support. The source of the bleeding was identified at the pulmonary artery proximal to the ligatures that had been placed. The bleeding was successfully controlled, and the patient responded to resuscitation measures by returning to normal sinus rhythm. After achieving hemodynamic and respiratory stability and managing the bleeding, it was decided to proceed with the surgery.

After the surgery, the patient was transferred to intensive care, intubated, sedated, mechanically ventilated, and hemodynamically unstable, with a high need for vasopressor support (norepinephrine 0.84 mcg/kg/min). Postoperative cardiac echocardiogram was normal. The patient showed decreased urine output and increased plasma potassium during the night, and the nephrologist on call performed a short dialysis session.

On the second day, the increased requirements of vasopressors (norepinephrine=1 mcg/kg/min, vasopressin=0.03 UI/min) and inotropes (dobutamine=7.14 mcg/kg/min), accompanied by decreased urine output, have driven to the decision to initiate fluid resuscitation guided by PiCCO parameters and to initiate continuous venovenous hemodiafiltration with continuous heparin anticoagulation. Relevant PiCCO parameters indicated a systemic vascular resistance index (SVRI) of 2895 (normal range 1700-2400 dyn.s.cm−5.m−2), extravascular lung water index (ELWI) of 4.7 (normal range 3-7 ml/kg), and stroke volume variation (SVV) of 19 (normal range ≤10%), with the administration of higher amounts of fluids in order to achieve optimal intravascular volume. RRT was discontinued after 24 hours as the patient's urine output recovered, inotropic support was ceased, and the vasoactive support doses were minimized. Subsequently, attempted ventilator weaning trials were unsuccessful due to patient-ventilator asynchrony. Successful extubation and return to normal neurological status were achieved only on the fifth day post-surgery.

Following the seventh day post-surgery, a CT scan revealed the expected thoracic changes following pneumonectomy, as well as bilateral temporal supratentorial petechiae without neurosurgical indication. Notably, there was no evidence of any neurological impairment.

On the ninth day post-surgery, the patient was transferred to the ward but later experienced clinical signs of pulmonary embolism (sudden and severe shortness of breath). The pulmonary CT angiogram revealed a blockage in the right superior lobar pulmonary artery. After being readmitted to the ICU, the patient received two doses of low-molecular-weight heparin (enoxaparin 8000 IU) per day to manage the pulmonary embolism, in addition to the previous treatment.

To provide a comprehensive understanding, Table 1 includes blood gas analysis and the most significant laboratory data of the patient at various moments during the first 24 hours of the treatment process and at the moment of discharge from the ICU. During the patient's stay in the ICU, a variety of therapeutic interventions were employed, encompassing the administration of crystalloid, electrolytes, albumin, antibiotics in consultation with an infectious disease specialist, prophylaxis for stress ulcers, analgesics, vitamins, bronchodilators, anticoagulants, sedatives, beta-blockers, inotropic and vasoactive agents, as well as nutritional support.

**Table 1:** Blood gas analysis. Intraoperative period. ICU stay day 1. At discharge from the ICU

Timeline	pH	paO_2 _(mmHg)	FiO_2_ (%)	paCO_2_ (mmHg)	SaO_2_ (%)	Lactate (mmol/L)	HCO_3_ (mmol/L)
After induction: one-lung ventilation							
IntraOP 1	7.2	176	60	60	98	0.8	23
IntraOP 2	7.1	250	60	76	99	2	23
Intraoperative hemorrhage starts							
IntraOP 3	7.1	182	60	74	99	3.8	20
After cardiopulmonary resuscitation							
IntraOP 4	7.1	222	100	55	99	7.7	17
IntraOP 5	7.2	218	100	34	99	6.9	16
ICU							
Immediately postOP	7.1	345	100	74	99	4.1	19
3 hours postOP	7.1	172	60	65	99	2.8	21
8 hours postOP	7.2	136	60	52	99	1.8	21
12 hours postOP	7.2	114	60	59	98	2.3	21
16 hours postOP	7.3	155	60	54	99	2.2	30
20 hours postOP	7.2	165	60	54	99	2.5	25
24 hours postOP	7.2	200	60	60	99	2.4	25
Before discharge from the ICU	7.4	146	21	32	98	1	24
Reference range	7.3-7.4	75-100	NA	35-40	95-99	0.6-2.2	22-32

Following a period of 12 days in the ICU, the patient was transferred to the ward. Subsequently, on the 19th day post-surgery, the patient was discharged in a favorable condition. Throughout the 18 months subsequent to the pneumonectomy, the patient consistently attended scheduled follow-up appointments at the thoracic surgery department in good health without any associated complications (Figure 5).

**Figure 3:**
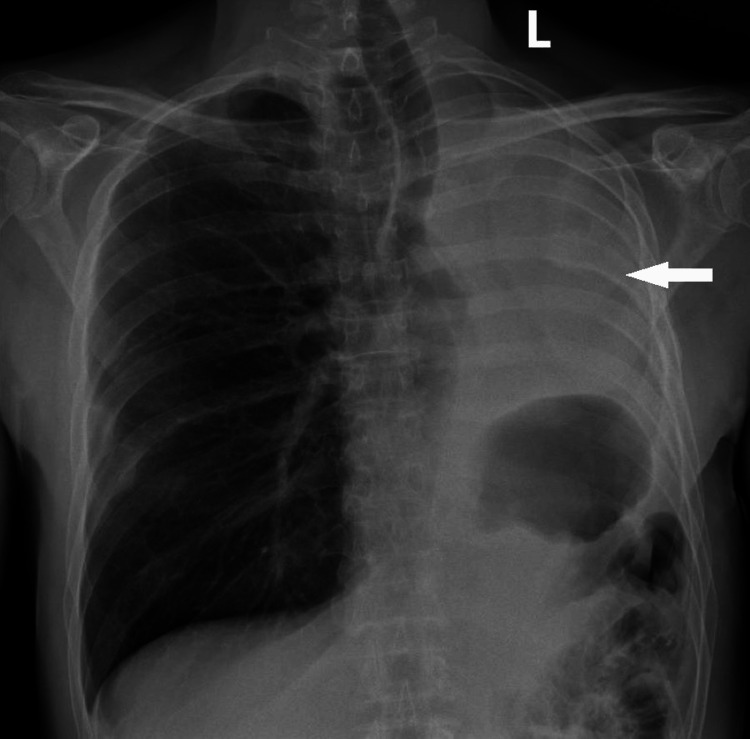
Five-month follow-up chest X-ray showing left side mediastinal shift with fibrinoid collection in the pneumonectomy cavity.

## Discussion

Thoracic surgery can be very challenging due to several considerations; one of the biggest challenges anesthesiologists face during thoracic surgery is managing respiratory function. Proper airway management is essential during thoracic surgery to prevent injury and ensure adequate oxygenation. Another major consideration during thoracic surgery is patient positioning. The patient may need to be positioned in such a way as to allow access to the surgical site, which can lead to hemodynamic complications such as hypotension or tachycardia. Pain management is also an important consideration during thoracic surgery. Bleeding is another potential complication during thoracic surgery due to the proximity of major blood vessels and the heart. Proper hemostasis techniques are necessary to minimize the risk of bleeding and prevent complications. Finally, patient factors such as age, comorbidities, and overall health status can also contribute to the complexity of thoracic surgery and anesthesia management. Overall, managing anesthesia during thoracic surgery requires careful attention to detail and a comprehensive understanding of the many factors that can contribute to complications. Despite the advances in the surgical field, intraoperative bleeding is not rare. There is a trend toward using minimally invasive procedures for pathologies (pulmonary, mediastinal, pancreatic, hepatic neoplasia, or hydatidosis) traditionally managed with open surgery. The reason for choosing video-assisted thoracoscopic surgery (VATS) over open surgery is that VATS results in less bleeding compared to open thoracotomy, with most bleeding events being due to vascular injury [14,15].

During thoracic surgery, the likelihood of experiencing cardiac arrest in the operating room is greater than 50% [16]. However, it's important to note that such occurrences are often remediable, resulting from reversible causes. Having a team of experienced medical professionals and advanced resources readily available in operating rooms is crucial to ensure the best possible outcomes in such situations [16] and offers the patient the best care that can be provided. In our case, the cause of the cardiac arrest was rapidly detected and controlled, open chest cardiac compressions were started early, and fast replacement of blood products, early defibrillation, and administration of vasopressor and positive inotropic agents with permissive hypotension led to the success of the resuscitation.

Acute kidney injury following thoracic surgery is about 15.1% and is associated with more significant morbidity and mortality [17]. After the surgery, the patient experienced a notable decline in urine output and increased serum potassium levels. Although there was no significant change in the urea and creatinine levels, it was evident that the patient's renal function was at risk. In order to prevent further damage to the kidneys and restore hemodynamic stability, we promptly initiated continuous venovenous hemodiafiltration. This intervention aimed to effectively filter and remove harmful substances from the patient's blood while maintaining appropriate fluid balance. By delivering this therapy early on, we were able to prevent the development of more serious renal complications and promote a faster recovery for the patient.

The incidence of deep vein thrombosis (DVT) after thoracic surgery is estimated to be between 0.4% and 51%. The incidence of pulmonary embolism is between 1% and 5%, and pneumonectomy is associated with a threefold increase in post-discharge venous thromboembolism (VTE) events compared to lobectomy [18]. SARS-CoV-2 infection increases the risk of blood clot formation and thrombotic complications, such as pulmonary embolism. This is due to hypercoagulable and pro-inflammatory states commonly encountered in this particular viral infection [19]. Recent or perioperative SARS-CoV-2 infection may independently increase the risk of postoperative VTE. This is because the underlying hypercoagulable state associated with the infection, combined with the surgical procedure, creates a higher risk of VTE [20]. Our patient experienced a pulmonary embolism with clinical impact nine days after the surgery. Although prophylactic anticoagulation and early mobilization were initiated, the patient still developed this condition. Therefore, it is essential to be alert for signs and symptoms of pulmonary embolism in these patients, such as sudden onset of shortness of breath, chest pain, and hemoptysis.

## Conclusions

Our case highlights the difficulties that can arise in managing a patient before, during, and after a radical pneumonectomy. Despite thorough preoperative preparation, this patient experienced various complications. The unique aspect of this case is the occurrence of a majority of complications commonly linked to major thoracic surgery within a single patient. Although there were difficulties to overcome, the patient's medical course went well. This positive outcome resulted from the surgical and anesthetic teams working harmoniously, aided by clear communication. This enabled prompt medical intervention and effective management throughout the perioperative period.
